# Cross‐sectional associations between healthy eating index and thyroid function in U.S. male Adults, NHANES 2007–2012

**DOI:** 10.1002/fsn3.3270

**Published:** 2023-02-14

**Authors:** Yiqiu Chen, Ting Han

**Affiliations:** ^1^ Department of Clinical Nutrition, Shanghai Tenth People's Hospital, School of Medicine Tongji University Shanghai China

**Keywords:** diet quality, healthy eating index, male Adults, NHANES, thyroid

## Abstract

Little is known about whether diet quality is associated with thyroid function. We aimed to examine the relationship between diet quality and thyroid function. Data were from the National Health and Nutrition Examination Surveys, 2007–2012. A total of 3603 males who were at least 20 years old and had dietary recall data were included in the analysis. Thyroid function was assessed by eight indexes, including total and thyroglobulin antibodies, thyroid peroxidase antibodies, free T4 and T3, total T4 and T3, Tg, and thyroid‐stimulating hormone. Multivariable linear regression, subgroup analyses, and interaction terms were employed to test the association between healthy eating index (HEI) and thyroid function. A total of 3603 male participants aged ≥20 years with an average age of 48.17 ± 0.51 years were enrolled. We found a negative association between HEI‐2010 and total T3 (β = −3.41; *p* = .01) and free T3 (β = −0.06; *p* = .01). In subgroup analyses, HEI‐2010 was negatively associated with TT3 in male participants aged <65 years old (β = −4.57; *p* < .01) and FT3 (β = −0.09; *p* < .001). Higher HEI‐2010 was inversely associated with lower total T3 and free T3. More well‐designed studies are still needed to validate the causal relationship between HEI and thyroid function.

## Graphical abstract

Little is known about whether diet quality is associated with thyroid function. We aimed to examine the relationship between diet quality and thyroid function. Multivariable linear regression, subgroup analyses, and interaction terms were employed to test the association between healthy eating index (HEI) and thyroid function. Higher HEI‐2010 was inversely associated with lower total T3 and free T3.

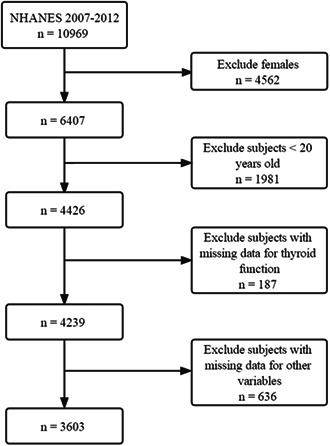

## 1 INTRODUCTION

The thyroid gland is a major endocrine gland that plays an important role in maintaining your weight, keeping you warm, and maintaining the function of your brain, heart, muscles, and other organs (Nilsson & Fagman, 2017; Ortiga‐Carvalho et al., 2016). The normal development, differentiation, and metabolism of almost every body cell depend on adequate thyroid hormone (TH) levels (Senese et al., 2019). The imbalance of THs (not necessarily pathology) may result in poorer cognitive outcomes, osteoporosis, cardiovascular disease, and varying degrees of goiter (Delitala et al., 2020; Londzin‐Olesik & Kos‐Kudła, 2020; Mullur et al., 2014; van Vliet et al., 2021; Yang et al., 2021).

Dietary factor is thought to play a role in the regulation of thyroid function (Brdar et al., 2021). Previous studies have indicated that TH levels are correlated with intake of multiple nutrients, including iodine intake, selenium, iron, and vitamin D (Kim et al., 2019; Niafar et al., 2016; O'Kane et al., 2018). Consumption of high‐glycemic index foods was positively associated with FT3 and FT4 levels, but negatively associated with TSH levels. However, the levels of FT3 and FT4 were negatively correlated with saturated fats and proteins in foods (Brdar et al., 2021).

Overall diet quality is critical when it comes to determining the health outcomes of diet since dietary intake is often combined to produce health outcomes (Schwingshackl et al., 2018; Wilson et al., 2016). The healthy eating index (HEI) indicates how closely dietary patterns match Dietary Guidelines for Americans and the food guide pyramid, which can be used to predict chronic diseases (Guenther et al., 2014). Dietary quality measured by HEI‐2015 total and component food scores significantly reduced osteoporosis risk in the NHANES (Fan et al., 2022). Based on the results of a meta‐analysis, the alternate HEI‐2010 was associated with a lower risk of depression (Lassale et al., 2019). HEI‐2010 is considered to be one of the reliable indexes for assessing diet quality.

Our study examined whether thyroid function is related to diet quality from a macro perspective using the HEI to determine whether men's diet quality is related to thyroid function. Therefore, determining the relationship between HEI‐2010 and thyroid function might bring new views on nutritional management of thyroid condition.

## 2 METHODS

The National Health and Nutrition Examination Survey (NHANES) survey collected information on demographics, health, and dietary intake in a nationally representative sample of 2‐year cycles through a complex, multistage probability design. Informed consent was provided to all participants as part of the NHANES protocol which has been approved by the National Center for Health Statistics Research Ethics Review Board.

**FIGURE 1:**
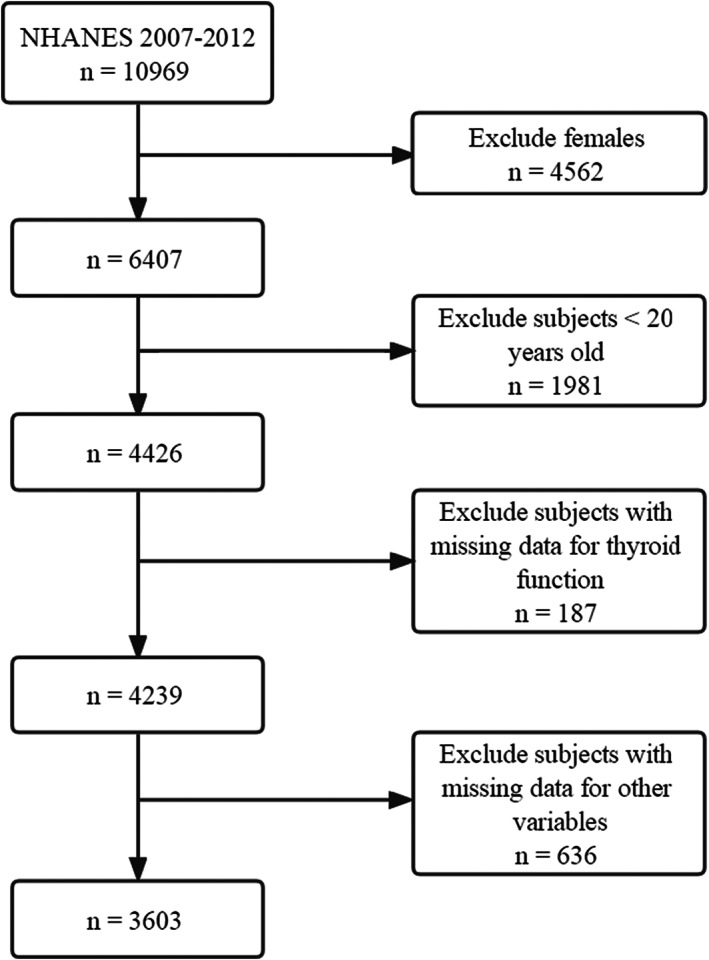
Selection process for the study sample based on the 2007–2012 National Health and Nutrition Examination Survey (NHANES).

An analysis of data from 3 cycles is presented (2007–2008, 2009–2010, and 2011–2012). Male adults aged 20 years and older were included in the study. Those whose thyroid function were unreliable were excluded (*n* = 187). The analysis also excluded those with missing data for other variables (*n* = 636). The descriptions are presented in more detail in Figure 1, and final sample size was 3603 male participants aged 20 years and older.

### 2.1 Assessment of diet quality

According to the HEI‐2010, diet quality is determined using both the vegetarian and vegan versions of the USDA Food Pattern as well as the omnivore version of the USDA Food Pattern. In HEI‐2010, there are 12 components, including 9 adequacy components (total fruit, whole fruit, total vegetables, greens and beans, whole grains, dairy, total protein foods, seafood and plant proteins, and fatty acids as the ratio of polyunsaturated and monounsaturated fatty acids to saturated fatty acids) and 3 moderation components (refined grains, sodium, and empty calories), with higher scores reflecting better diet quality (Guenther et al., 2013). With the simple HEI scoring algorithm, the present analysis calculated diet quality for each person based on mean dietary intakes from two 24‐hour recalls. Scores from the 12 components are added up for HEI‐2010, which has a maximum score of 100.

### 2.2 Measurement of thyroid outcomes

In this study, thyroid function parameters were examined for total and free thyroxine (T4), total and free triiodothyronine (T3), thyroglobulin (Tg), thyroglobulin antibodies, thyroid peroxidase antibodies, and thyroid‐stimulating hormone (TSH). A detailed explanation of the procedure for collecting and processing specimens was provided in the NHANES Laboratory/MedicalTechnologists Procedures Manual (LPM).

### 2.3 statistical analysis

We used R (version 4.1.2) to perform all statistical analyses. A *p* Value of .05 was considered statistically significant in all reported probabilities (*p* Values). Where appropriate, sampling weights were used to account for the complex multistage design of the survey. In this study, categorical variables are expressed as percentages, while continuous variables are expressed as mean with standard deviation. Based on quartiles, HEI‐2010 was categorized (quartile 1: <25th percentile, quartile 2: ≥25th to 50th percentile, quartile 3: ≥50th to 75th percentile, quartile 4: ≥75th percentile). Differences in characteristics were tested between groups by a chi‐square test for categorical variables and by a one‐way analysis of variance for continuous variables. The association between HEI‐2010 and thyroid function was investigated using multivariable linear regressions. Also, subgroup analyses of BMI and age categories were performed and all covariates except itself were adjusted. An interaction term was added to test the heterogeneity of the associations.

## 3 RESULTS

There were 3603 male adults in the sample, with an average age of 48.17 ± 0.51 years. Table 1 shows the distributions of demographic characteristics overall and by HEI quartiles. In the upper quartiles of HEI, men had better educational qualifications, had higher household incomes, were more likely to be married, and were more likely to never smoke. In terms of age, race, education, marital status, poverty income ratio, smoking status, and energy intake, there was a significant difference among the HEI quartiles (*p* < .05). There were 73.2% participants being overweight or obese. In terms of BMI and urinary iodine, no significant differences across HEI‐2010 quartiles were observed. For thyroid parameters, the statistical significance was observed in TT3, FT3, TT3/TT4 ratio, and FT3/FT4 ratio, which among all participants were 114.73 ± 0.78 ng/dL, 3.2 ± 0.01 pg/mL, 0.33 ± 0.002, and 15.50 ± 0.12, respectively.

**TABLE 1:** Demographic characteristics and thyroid parameters of male adults 20 years and older (*n* = 3603) by quartile of Healthy Eating Index‐2010 in National Health and Nutrition Examination Surveys 2007–2012.

Variable	HEI^c^‐2010 quartile				*p*‐Value
	Q1	Q2	Q3	Q4	
*N*	901	901	900	901	
Age (years), Mean ± SD^a^	42.35 ± 0.56	44.13 ± 0.66	47.73 ± 0.88	67.15 ± 0.32	**<.0001**
Race/ethnicity (%)					
White	71.47	68.03	69.88	72.32	**.01**
Black	11.63	10.37	8.26	7.89	
Mexican	8.64	9.85	9.68	6.70	
others	8.25	11.75	12.17	13.09	
BMI^b^ (kg/m^2^) (%)					
<25	25.98	28.06	27.62	26.25	.18
25–30	36.22	38.26	39.11	43.82	
≥30	37.80	33.68	33.27	29.93	
Education (%)					
Less than high school	21.94	21.44	19.75	12.96	**<.0001**
High school	33.49	25.88	22.16	18.40	
More than high school	44.58	52.69	58.09	68.64	
Marital status (%)					
Never married	22.49	23.21	16.07	17.32	**<.001**
Divorced/Widowed/Separated	14.48	14.38	11.42	10.06	
Married/Partner	63.03	62.41	72.51	72.62	
PIR^d^ (%)					
<1	15.66	14.77	10.79	9.92	**.002**
≥1	84.34	85.23	89.21	90.08	
Smoking status (%)					
Never	43.20	46.18	46.52	50.66	**<.0001**
Past	23.09	25.52	32.80	36.78	
Current	33.71	28.30	20.69	12.56	
Energy intake (kcal), Mean ± SD	2794.94 ± 44.23	2665.56 ± 52.60	2514.73 ± 39.00	2399.37 ± 42.11	**<.0001**
Urinary iodine (ug/L), Mean ± SD	245.85 ± 13.71	251.94 ± 17.86	254.06 ± 17.01	257.21 ± 18.75	.95
Thyroglobulin antibodies (IU/mL), Mean ± SD	9.75 ± 2.31	7.24 ± 2.86	4.32 ± 1.54	5.17 ± 1.97	.17
Thyroid peroxidase antibodies (IU/mL), Mean ± SD	14.15 ± 2.46	10.71 ± 2.58	13.28 ± 3.61	12.98 ± 2.65	.82
Total T4 (μg/dL), Mean ± SD	7.60 ± 0.07	7.54 ± 0.07	7.56 ± 0.07	7.63 ± 0.06	.76
Total T3 (ng/dL), Mean ± SD	118.62 ± 1.28	116.03 ± 1.17	113.24 ± 0.87	110.69 ± 0.92	**<.0001**
Free T4 (pmol/L), Mean ± SD	10.18 ± 0.09	10.16 ± 0.11	10.22 ± 0.10	10.40 ± 0.10	.11
Free T3 (pg/mL), Mean ± SD	3.36 ± 0.02	3.30 ± 0.02	3.26 ± 0.02	3.19 ± 0.02	**<.0001**
Free T3/Free T4	0.34 ± 0.003	0.33 ± 0.004	0.33 ± 0.003	0.32 ± 0.003	**<.0001**
Total T3/Total T4	15.92 ± 0.20	15.78 ± 0.17	15.41 ± 0.18	14.84 ± 0.14	**<.0001**
TSH (mIU/L), Mean ± SD	1.91 ± 0.06	1.88 ± 0.09	1.95 ± 0.06	1.95 ± 0.05	.75
Thyroglobulin (ug/L), Mean ± SD	13.82 ± 0.92	13.19 ± 0.69	14.90 ± 1.87	12.11 ± 0.54	.13

*Note*: Values are survey‐weighted percentages. Values in bold indicate significant at *P* < 0.05.

^a^ SD = standard deviation.

^b^ BMI = body mass index.

^c^ HEI = healthy eating index.

^d^ PIR = poverty income ratio.

The associations between HEI‐2010 and thyroid function are presented in Table 2. HEI was negatively correlated with total T3 (*p*‐trend <.001), free T3 (*p*‐trend <.001), FT3/FT4 ratio (*p*‐trend <.001), and TT3/TT4 ratio (*p*‐trend = .004) in Model 1 adjusted for age, race, and urinary iodine. The highest HEI‐2010 quartile was significantly associated with decreased TT3 (β=: −5.29; 95% CI: −7.97, −2.61; *p* < .001), FT3 (β=: −0.08; 95% CI: −0.13, −0.04; *p* < .001), FT3/FT4 (β=: −0.01; 95% CI: −0.02, −0.01; *p* < .001), and TT3/TT4 (β=: −0.60; 95% CI: −1.05, −0.16; *p =* .01). The inverse association between HEI and total T3 (*p*‐trend = .01) or free T3 (*p*‐trend = .02) had been attenuated but remained significant in the fully adjusted model (Model 2). The HEI‐2010 (highest versus lowest) was negatively associated with TT3 (β: −3.41; 95% CI: −6.06, −0.77; *p* = .01) and FT3 (β: −0.06; 95% CI: −0.10, −0.02; *p* = .01).

**TABLE 2:** Association between HEI^g^‐2010 total score and thyroid function among U.S. adult men in NHANES from 2007 to 2012.

Quartiles of HEI‐2010	TT3 (ng/dL)^a^	FT3 (pg/mL)^b^	FT3/FT4^c^	TT3/TT4^d^
	Model 1 β (95%^f^ CI) *p*‐value^e^			
Q1	Ref	Ref	Ref	Ref
Q2	−2.14 (−4.61, 0.34) .09	−0.04 (−0.07, −0.01) **.01**	0 (−0.01, 0.01) .57	‐0.01 (−0.41, 0.40) .97
Q3	−3.75 (−6.40, −1.10) **.01**	−0.04 (−0.09, 0.01) .08	0 (−0.01, 0.00) .51	−0.17 (−0.57, 0.23) .38
Q4	−5.29 (−7.97, −2.61) **<.001**	−0.08 (−0.13, −0.04) **<.001**	−0.01 (−0.02, −0.01) **<.001**	−0.60 (−1.05, −0.16) **.01**
*p* for trend	**<.001**	**<.01**	**.001**	**.004**
	Model 2 β (95% CI) *p*‐value			
Q1	Ref	Ref	Ref	Ref
Q2	−1.45 (−3.88, 0.98) .23	−0.03 (−0.07, 0.00) .07	0 (−0.01, 0.01) .92	0.1 (−0.31, 0.50) .63
Q3	−2.5 (−5.08, 0.08) .06	−0.02 (−0.07, 0.02) .27	0 (−0.01, 0.01) .89	−0.01 (−0.43, 0.41) .96
Q4	−3.41 (−6.06, −0.77) **.01**	−0.06 (−0.10, −0.02) **.01**	−0.01 (−0.02, 0.00) **.02**	−0.36 (−0.83, 0.11) .13
*p* for trend	**.01**	**.02**	.06	.09

*Note*: Values in bold indicate significant at *P* < 0.05.

^a^ TT3, total triiodothyronine.

^b^ FT3, free triiodothyronine.

^c^ FT4, free thyroxine.

^d^ TT4, total thyroxine.

^e^ Model 1: age, race, and urinary iodine were adjusted. Model 2: age, race, smoking status, energy intakes, education level, BMI, marital status, PIR, and urinary iodine were adjusted.

^f^ CI, confidence interval.

^g^ HEI, Healthy Eating Index.

In Table 3 and Table 4, further analyses were conducted stratified by BMI and age categories, as the two variables might be potential effect modifiers. The original relationship among HEI and total T3, free T3, FT3/FT4 showed stronger negative associations in the <65 year group after subgroup analysis. According to the subgroup analysis, age was the interactive factor in the correlation between HEI and TT3 (*p* for interaction = .02), as well as FT3 (*p* for interaction = .006). Table 3 indicates that the association between HEI and TT3 (β = −4.57, 95% CI: −7.57 to −1.57, *p* < .01, *p* for trend = .004), FT3 (β = −0.09, 95% CI: −0.14 to −0.05, *p* < .001, *p* for trend <.001) for quartile 4 was more significant in the male subjects aged <65 years. The ratio of FT3/FT4 also showed a similar relationship with HEI. However, in the fully adjusted model, we were unable to observe interaction effects between BMI group and HEI‐2010 on thyroid function (*p* for interaction >.05).

**TABLE 3:** Subgroup analysis of association between HEI^a^‐2010 and thyroid function stratified by BMI^b^ groups.

Quartiles of HEI‐2010	TT3 (ng/dL)^c^	FT3 (ng/dL)^d^	FT3/FT4^e^	TT3/TT4^f^
	Subgroup analysis stratified by BMI^g^			
	Normal weight			
Q1	Ref	Ref	Ref	Ref
Q2	−4.37 (−10.30, 1.57) .14	−0.01 (−0.09, 0.07) .80	0.01 (0.00, 0.02) .09	0.16 (−0.52, 0.84) .63
Q3	−7.06 (−11.67, −2.45) **.004**	−0.04 (−0.12, 0.04) .33	0 (−0.02, 0.01) .74	−0.74 (−1.53, 0.05) .06
Q4	−2.05 (−6.73, 2.63) .38	−0.04 (−0.13, 0.04) .32	−0.01 (−0.03, 0.00) .14	−0.38 (−1.26, 0.49) .38
*p* for trend	.27	.27	.07	.19
	Overweight			
Q1	Ref	Ref	Ref	Ref
Q2	−2.31 (−7.53, 2.92) .38	−0.1 (−0.16, −0.04) **.002**	−0.01 (−0.02, 0.00) .06	0.1 (−0.40, 0.60) .69
Q3	−1.7 (−5.50, 2.09) .37	−0.06 (−0.14, 0.02) .13	0 (−0.01, 0.01) .86	0.5 (−0.02, 1.01) .06
Q4	−3.7 (−8.03, 0.64) .09	−0.09 (−0.16, −0.01) **.02**	‐0.01 (−0.02, 0.01) .37	−0.07 (−0.66, 0.52) .82
*p* for trend	.09	.06	.67	.99
	Obese			
Q1	Ref	Ref	Ref	Ref
Q2	1.66 (−3.26, 6.57) .50	0.02 (−0.04, 0.08) .46	0.01 (−0.01, 0.02) .33	0.06 (−0.74, 0.86) .88
Q3	−0.37 (−5.48, 4.75) .89	0.02 (−0.05, 0.09) .54	0.01 (−0.01, 0.02) .24	−0.07 (−0.94, 0.79) .86
Q4	−4.48 (−9.42, 0.47) .07	−0.04 (−0.11, 0.03) .23	−0.01 (−0.02, 0.00) .16	−0.74 (−1.35, −0.12) **.02**
*p* for trend	.07	.32	.20	**.03**
*p* for interaction	.65	.91	.28	.27

*Note*: Values in bold indicate significant at *P* < 0.05.

^a^ HEI, Healthy Eating Index.

^b^ BMI, body mass index.

^c^ TT3, total triiodothyronine.

^d^ FT3, free triiodothyronine.

^e^ FT4, free thyroxine.

^f^ TT4, total thyroxine.

^g^ Subgroup analysis results were adjusted for age, race, smoking status, energy intakes, education level, marital status, PIR, and urinary iodine.

**TABLE 4:** Subgroup analysis of association between HEI^a^‐2010 and thyroid function stratified by age group.

Quartiles of HEI‐2010	TT3 (ng/dL)^b^	FT3 (ng/dL)^c^	FT3/FT4^d^	TT3/TT4^e^
	Subgroup analysis stratified by age <65^f^			
Q1	Ref	Ref	Ref	Ref
Q2	−1.36 (−4.16, 1.45) .33	−0.03 (−0.07, 0.01) .13	0 (−0.01, 0.01) .72	0.17 (−0.28, 0.61) .45
Q3	−2.88 (−5.79, 0.03) .05	−0.04 (−0.09, 0.01) .09	0 (−0.01, 0.01) .89	−0.04 (−0.51, 0.43) .87
Q4	−4.57 (−7.57, −1.57) **<.01**	−0.09 (−0.14, −0.05) **<.001**	−0.01 (−0.02, 0.00) **.03**	−0.51 (−1.03, 0.00) .05
*p* for trend	**.004**	**<.001**	**.04**	**.04**
	≥65			
Q1	Ref	Ref	Ref	Ref
Q2	0.3 (−4.04, 4.64) .89	−0.05 (−0.13, 0.03) .18	−0.01 (−0.02, 0.01) .54	−0.33 (−1.18, 0.52) .43
Q3	0.27 (−4.98, 5.52) .92	−0.01 (−0.10, 0.09) .91	0.01 (−0.01, 0.02) .48	−0.04 (−0.83, 0.74) .91
Q4	1.12 (−3.85, 6.10) .65	−0.03 (−0.10, 0.03) .32	−0.01 (−0.02, 0.01) .37	−0.21 (−0.98, 0.55) .57
*p* for trend	.65	.67	.50	.80
*p* for interaction	**.02**	**.006**	.22	.14

*Note*: Values in bold indicate significant at *P* < 0.05.

^a^ HEI, Healthy Eating Index.

^b^ TT3, total triiodothyronine.

^c^ FT3, free triiodothyronine.

^d^ FT4, free thyroxine.

^e^ TT4, total thyroxine.

^f^ Subgroup analysis results were adjusted for race, smoking status, energy intakes, education level, BMI, marital status, PIR, and urinary iodine.

## 4 DISCUSSION

This study examined whether thyroid function was associated with general eating patterns, as measured by the HEI, using multivariable models that were adjusted for confounding factors. The nationally representative survey of U.S. male adults showed a negative relationship between HEI‐2010 and TT3, FT3, and a negative association between HEI‐2010 and FT3/FT4, TT3/TT4 when the ratios of thyroid function measures were evaluated.

Although there is no doubt that iodine intake significantly affects thyroid function, several epidemiological studies have found that diet intake (not related to iodine) could also play a role. It was found by Stella Iacovides et al. that a ketogenic diet decreased T3 levels and increased T4 levels, while a high‐carbohydrate, low‐fat diet did not affect thyroid function (Iacovides & Maloney, 2022). Alessio Basolo et al. reported that a high‐protein overfeeding led to plasma TSH, FT3, and FT4 decrease, while a low‐protein overfeeding resulted in a reduction in plasma TSH and increase in plasma FT3 (Basolo et al., 2019).

Studies about the relationship between diet quality and thyroid function are still scarce. A cross‐sectional study indicated that higher adherence to Mediterranean diet (Med‐Diet) was found to be negatively related to free T3 and T4 in a cohort of overweight/obese subjects (Zupo & Castellana, 2020). Stavroula Lambrinakou et al. found that the frequency of non‐home‐made meal consumption was positively related to T4. T3 level was confirmed to be positively correlated with vegetables consumption cooked with olive oil, but negatively relationship with consumption of whole grain (Lambrinakou et al., 2017). Another study observed a 10‐point increase in HEI associated with 0.6% reduced total T4 among U.S. adults and 0.5% reduced total T4 among male adults (Melough et al., 2022). Consequently, a balanced diet is an important component of maintaining a healthy thyroid gland function.

A recent study by Roberta Zupo et al. examined the relationship between skeletal muscle mass and TH levels among subjects with overweight and obesity (Zupo et al., 2020). Nevertheless, subgroup analysis stratified by BMI showed no statistically significant increase between THs and higher HEI in obese subjects. The interaction term test also found no significant difference between HEI and thyroid function among different BMI groups, indicating that the heterogeneities among different subgroups were not statistically significant. As this study indicated, older age had a relatively higher predicted HEI‐2010 score. Diet quality increased by age among male participants, which was consistent with results in the study for general adult population (Hiza et al., 2013). Compared with the lowest education and income categories, significantly higher HEI‐2010 scores were observed in the highest categories in our analysis. Age‐stratified subgroup analysis indicated that the negative association between HEI and thyroid function became more evident in participants aged <65 years old. Since older adults have become more health conscious as they age and have improved their diets, they might be more motivated to have a healthy diet and a higher HEI total score.

This study has some strengths. In data collection and reporting, NHANES employed standardized and well‐controlled protocols. Second strength was using universalistic data from a broad representative sample from the U.S. adult population. Moreover, the NHANES, which is a comprehensive dataset, includes important covariates such as demographics. Our study does have certain restrictions, though. In terms of dietary patterns, the data used for HEI calculation were collected from a 24‐hour dietary recall, not necessarily representative of an individual's habitual diet. Due to the uncertain impact of pregnancy and menstruation on thyroid function in women, we excluded the female participants from our study. As our study is a cross‐sectional analysis, we cannot establish causation. To examine the relationship between chronic eating habits and their long‐term impact on thyroid function, a longitudinal cohort study is required.

## 5 CONCLUSION

This study showed a significant association of higher HEI‐2010 score and decreased total T3 in male adults, and the association might become stronger in men <65 years, based on data from NHANES 2007–2012. Our study may indicate that nutritional management might be necessary to ensure normal thyroid function. However, this study is just cross‐sectional observational, prospective cohort studies, or randomized controlled trials are warranted to explore the causal relationship between HEI and thyroid function.

## CONFLICT OF INTEREST STATEMENT

The authors declare that they have no competing interests.

## ETHICS APPROVAL STATEMENT

The Centers for Disease Control and Prevention and the Research Ethics Review Board at the National Center for Health Statistics conducted the consents and have received ethical approval.

## Data Availability

The specific dataset associated with this study will be made available by the corresponding author upon reasonable request.
